# A malignant spiradenoma of the forehead: a case report and literature review

**DOI:** 10.1093/omcr/omae016

**Published:** 2024-03-25

**Authors:** Mohamed Badie Ahmed, Khaled E Elzawawi, Ayda AlHammadi, Aysha Al-Malki, Mahir Petkar, Salim Al Lahham, Abeer Alsherawi

**Affiliations:** Plastic Surgery Department, Hamad General Hospital, Hamad Medical Corporation, Doha, Qatar; College of Medicine, QU Health, Qatar University, Doha, Qatar; College of Medicine, QU Health, Qatar University, Doha, Qatar; Department of Dermatology and Venereology, Hamad Medical Corporation, Doha, Qatar; Department of Dermatology and Venereology, Hamad Medical Corporation, Doha, Qatar; Department of Laboratory Medicine and Pathology, Hamad Medical Corporation, Doha, Qatar; Plastic Surgery Department, Hamad General Hospital, Hamad Medical Corporation, Doha, Qatar; Plastic Surgery Department, Hamad General Hospital, Hamad Medical Corporation, Doha, Qatar

## Abstract

Spiroadenomas, arising from sweat glands, are rare benign skin tumors primarily found as solitary nodules on the head, neck, and trunk. The malignant subtype, Spiradenocarcinoma (MSA), originating from benign spiradenomas, is extremely rare and occurs mainly in individuals over 50. MSA exhibits aggressiveness with higher metastasis rates and lower survival rates. Surgical excision is the standard management, supported by imaging modalities like MRI, CT-scan, and ultrasound. We present a 69-year-old female with a medical history of diabetes, hypertension, and dyslipidemia who presented in 2014 with multiple swellings on the forehead and left arm. Initial excisions revealed capillary hemangioma lesions. Subsequent visits involved the excision of further facial and body lesions, with some identified as intradermal nevi. In 2022, she presented to plastic surgery clinic with forehead swelling. The biopsy showed MSA lesion with involved margins. Thus, subsequent re-excision was carried out. One year later, she came with recurrent forehead swelling. Excision and direct closure of the lesion showed involvement of part of the subcutaneous tissue (fat globule) showing residual/recurrent MSA that is very close to nerve trunks. Malignant Spiradenomas (MSAs) usually arise from benign spiradenomas. Therefore, consideration is given to preemptive removal of these tumors due to their potential evolution. The primary treatment approach involves surgery, with a focus on wide local excision and a minimum margin of 1 cm to diminish the risk of metastasis. Vigilant follow-up is essential to promptly identify any recurrences or spreading.

What’s already known about this topic?

- Malignant spiradenoma is an exceedingly uncommon malignant skin adnexal tumor.
- The primary approach is surgical, involving wide local excision with a margin of at least 1 cm.

What does this report add?

- Highlights the importance of suspecting these lesions early to avoid local metastasis.
- Shed light on the importance of follow-up visits to be able to monitor the lesion progression or recurrence after excision.

## INTRODUCTION

Spiroadenomas are rare, usually benign skin tumors that arise from sweat glands [1, 2]. Spiroadenomas are usually present as solitary nodules, yet they rarely also present as multiple nodules [2]. Spiroadenomas usually emerge in the head, neck, and trunk, however, there have been case reports on spiroadenomas growing on the breasts [3]. The exact cause of Spiroadenomas is still unknow, still it is thought that Spiroadenomas are the result of a defect in the CYLD tumor suppressor gene on chromosome 16. The rarer malignant subtype displays increased rates of mitosis, necrosis, nuclear atypia, and pleomorphism [1]. Benign Spiroadenomas are quite rare tumors, and the malignant subtype is even more exceedingly uncommon.

Spiradenocarcinoma, also known as Malignant SpirAdenoma (MSA), is an exceedingly uncommon malignant skin adnexal tumor. Dabska et al. reported the first case in 1971 [4]. The tumor usually originates from benign spiradenoma, however, few cases developed malignant immediately. MSA usually occurs in patients older than 50 with an average of 59 years [5]. There have been more than 90 reported cases in the literature, nevertheless MSA remains an extremely rare tumor [6]. Surgical excision is the usual recommended management for MSA. Although rare, MSA usually tends to be more aggressive tumors with higher rates of metastasis and lower survival rates [7]. MRI, CT-scan, and ultrasound are the recognized imaging modalities used to check for any metastasis whether local or distant metastasis. In case of metastasis, the use of chemotherapy and radiotherapy along with local surgical resection of the tumor are advised [1, 7].

## CASE PRESENTATION

This is a 69-year-old female patient with a history of diabetes mellitus, hypertension, and dyslipidemia, who presented to the plastic surgery clinic in 2014 with multiple swellings on the forehead and left arm. The excision of two facial lesions and one lesion on the left arm revealed capillary hemangioma lesions. A year later, in 2015, the patient returned to the plastic clinic with multiple lesions on the face, chest, and trunk. Excisional biopsy and direct closure of eight facial lesions and several other body lesions indicated that some of the facial lesions were intradermal nevi. In 2019, the patient visited the emergency department, complaining of pain and swelling on the right side of the forehead that had been growing for two years. The patient was referred to plastic surgery but missed several appointments.

**Figure 1:**
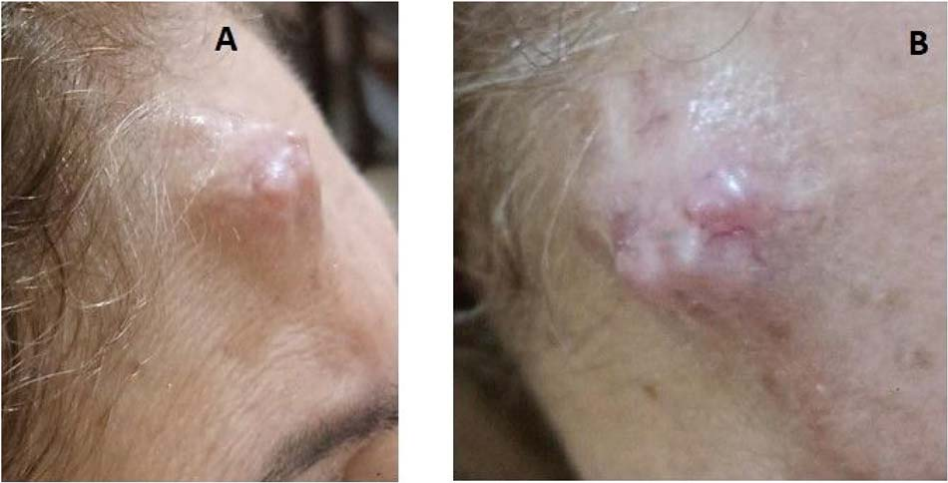
Preoperative image in 2022 showing the right forehead lesion.

In 2022, the patient returned to the plastic surgery clinic, complaining of a large lesion on the forehead (Fig. 1). A Computed Tomography (CT) scan of the face revealed a fairly well defined right frontal subcutaneous lesion containing central areas of cystic density measuring 1.0 × 1.5 × 1.6 cm. moreover, heterogenous mainly peripheral post contrast enhancement was noted along with absence of destruction of underlaying bone, intracranial extension or significant intracranial abnormality. Soft tissue ultrasound (U.S.) indicated an ill-defined, complex, heterogeneous structure on the right forehead (the area of swelling) measuring approximately 17.2 × 10 × 12.7 mm, with minimal vascularity observed in color doppler images.

**Figure 2:**
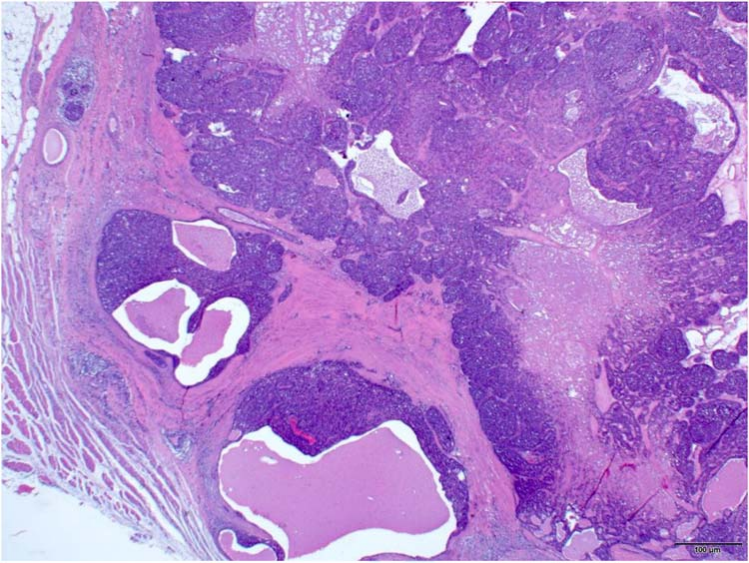
Irregular nodules of solid/cystic malignant neoplasm extending into the subcutaneous tissue (H and E × 4).

**Figure 3:**
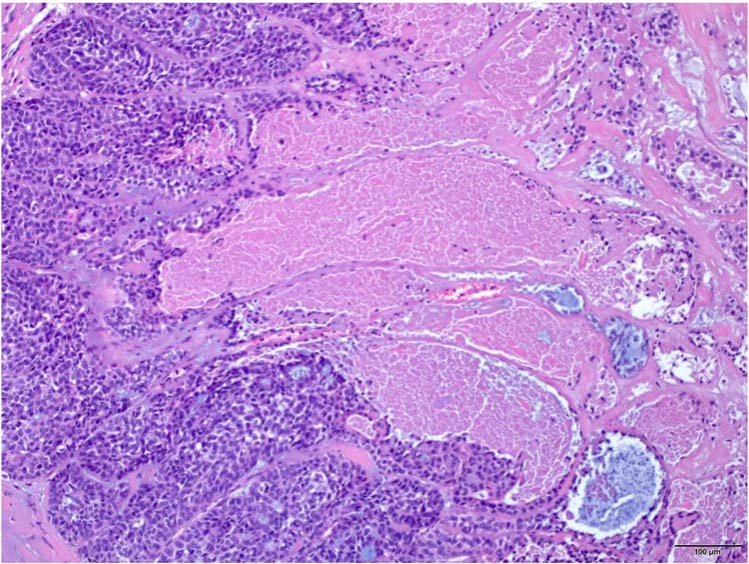
The tumor exhibits variable areas of necrosis (H and E × 10).

**Figure 4:**
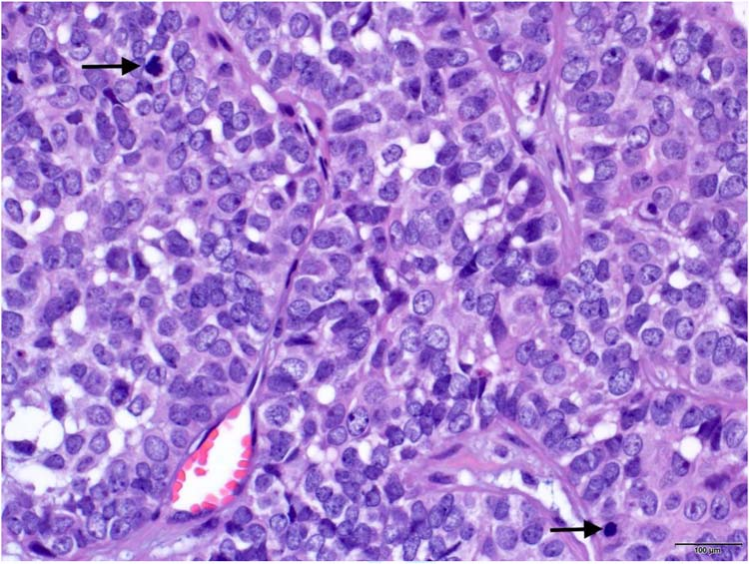
High power view showing nuclear hyperchromasia, nuclear pleomorphism and frequent mitosis (arrows) (H and E × 40).

**Figure 5:**
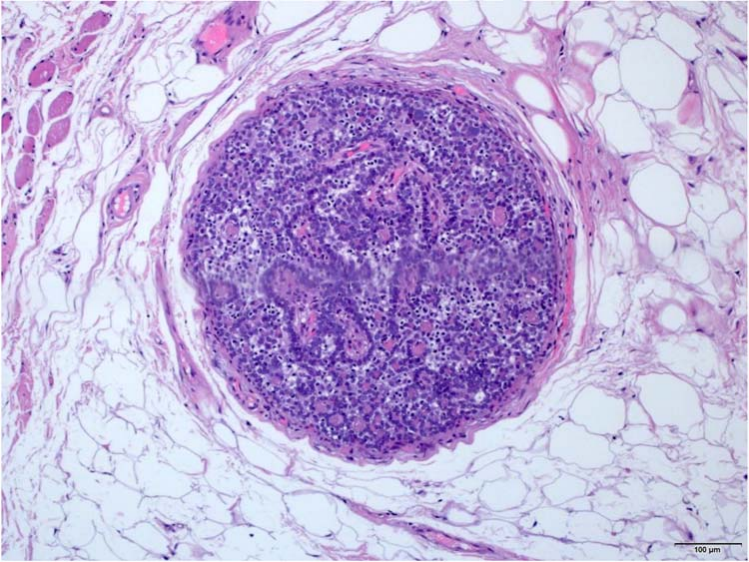
The periphery of the tumor reveals small nodules that are reminiscent of benign spiradenoma (H and E × 20).

**Figure 6:**
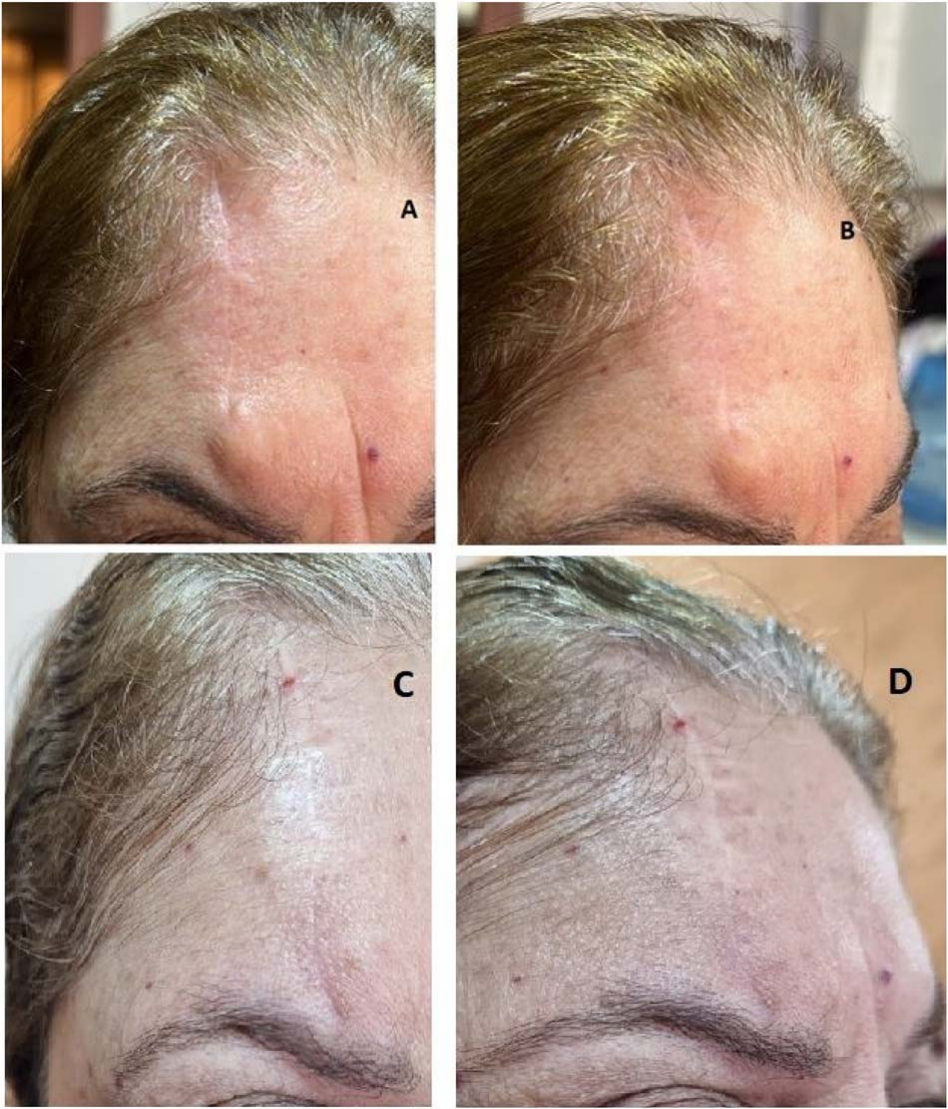
(**A** and **B**) Postoperative images after the first excision of the lesion with positive margins done in 2022. (**C** and **D**) Postoperative images after the excision recurrent swelling in 2023.

**Figure 7:**
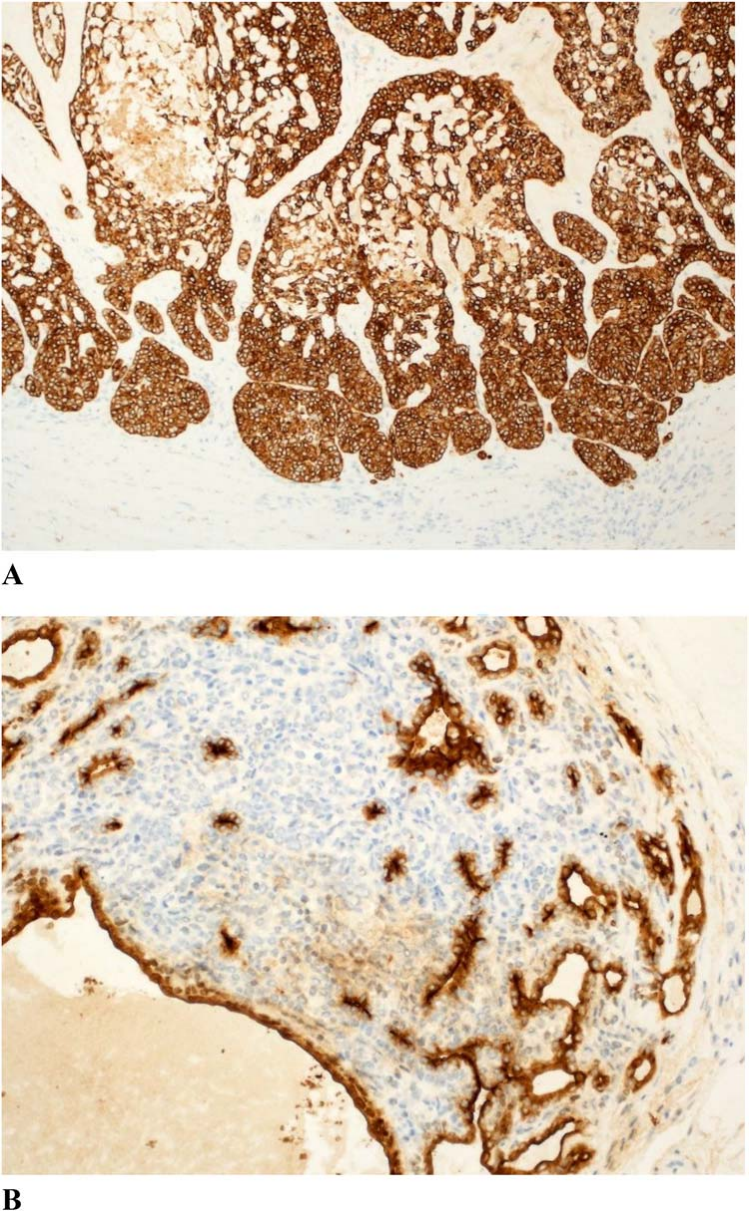
(**A**) The tumor shows diffuse and strong CK7 positivity. (**B**) EMA is focally positive.

An excisional biopsy of the swelling was performed under local anesthesia, and the surgical pathology report confirmed the diagnosis of malignant spiradenoma (Figs 2–5), which extended to the deep margin and was less than 1 mm from the peripheral margin (Fig. 6A and B). Immunohistochemistry showed the tumor was diffusely positive for CK7 (Fig. 7A), and was focally EMA positive (Fig. 7B). Subsequently, a re-excision with a 7 mm safety margin and direct closure was carried out, revealing residual MSA, less than 0.5 mm from the deep surgical margin. The patient was screened for metastasis, revealing bilateral infraclavicular heterogenous structures suspicious for lymph nodes. These structures were biopsied and found to be negative for malignant cells. Consequently, the patient was referred to medical oncology and started on radiotherapy.

One year later, in 2023, she presented back with a recurrent right forehead swelling. A skin punch biopsy was negative for dysplasia or malignancy. Excision and direct closure of the swelling showed skin with dermal scarring and extensive foreign body type granulomatous reaction, completely excised and negative for malignancy (Fig. 6C and D). However, part of the subcutaneous tissue (fat globule) showed residual/recurrent malignant spiradenoma very close to nerve trunks.

The skin cancer multidisciplinary team (MDT) recommended continuing the treatment with medical oncology and radiotherapy teams.

## DISCUSSION

MSA is a rare adnexal tumor that could pose both a diagnostic and a therapeutic challenge due to the paucity of published data regarding this entity. To date, there are no consensus nor clear guidelines for the management of MSA, and therapeutic options are obtained from published case reports or case series [8]. The incidence of this rare entity was estimated to be 0.07 cases per million. It is more common in the older population, with a median age at diagnosis of 63 years, and without a predilection for any specific gender. It could be associated with the autosomal dominant Brooke-Spielger syndrome [6]. The tumor would classically present as an abrupt growth of a long-standing indolent, painless nodule. Sometimes, pain and ulceration are associated features, which could be the main driver for seeking medical advice [8]. The time between appearance of the benign lesion and malignant transformation is variable, ranging from 6 months to 70 years, but it is usually around 20–30 years on average [9]. The diagnosis is confirmed based on histological analysis. In most cases, it requires the identification of the malignant component from a pre-existing benign lesion. If the tumor appeared de novo, histological analysis could be challenging and confused with other distant metastases or other carcinomas [8]. Other primary adnexal carcinomas, such as porocarcinoma, malignant cylindroma and malignant hidradenoma, may be difficult to distinguish from a malignant spiradenoma microscopically. While there are subtle histological differences between these entities, the clue to their diagnoses lies in the identification of the benign counterparts adjacent or close to the malignant tumor, as seen in our case. Additionally, porocarcinoma will usually display an in-situ component, which is not seen in malignant spiradenoma [10].

As per world health organization (WHO), there are two main histologic patterns for MSA; one shows a continuous transformation from benign to malignant neoplasm which is usually an important clue to the diagnosis; the other has the malignant transition adjacent to the spiradenoma without structural or cytological changes [5]. Histologically, spiradenomas are typically dermal nodules composed of multiple, well circumscribed, dense nests of basophilic cells. The cells are arranged in cords with two classic cell populations: small, peripheral cells with hyperchromatic nuclei and scanty cytoplasm, and larger, inner cells with vesicular nuclei. Benign spiradenomas average 0 to 2 mitotic figures per high-power field (HPF) [9].

Malignant transformations are characterized by a transition between benign and malignant areas with increased mitotic figures (mitotic rate was 8-20/HPF). Atypia, pleomorphism, ulceration, necrosis and hyperchormasia are features of the malignant lesions, observed depending on the grade of the tumor [9]. Based on the degree of atypia, MSAs are further classified into low-grade and high-grade neoplasms. Low-grade neoplasms are characterized by loss of dual cell population. They are typically composed of cells that appear monotonous and basaloid in nature, showing mild to moderate cytological atypia and a slight increase in mitotic activity [11, 12]. Some Low-grade neoplasms express clear cell change and squamoid features [12]. In high grade neoplasms, the transition from benign to malignant is usually more abrupt and the tumor shows severe cytological atypia, nuclear pleomorphism, necrosis and atypical mitotic activity. The high-grade malignant lesions could show a wide range of features including clear cell, oncocytic and mucinous change [11, 12]. Immunohistochemically, MSA express cytokeratins including EMA and CEA. MYB expression, which is usually positive in benign spiradenomas is lost in the malignant counterpart [12].

Regarding treatment, the primary approach for MSA is surgical, involving wide local excision with a margin of at least 1 cm. Local recurrence rates are around 20%, with 75% detected within the first 12 months of follow-up. Distant metastasis occurs in 16.7% to 37.4% of cases, commonly to regional lymph nodes (53.1%), lungs (34.7%), and bone (26.5%). Routine lymph node dissection is uncertain, but excision is recommended in cases of positive lymph nodes. Overall, a multidisciplinary approach is very important for accurate diagnosis and management of MSA [5, 6, 8, 13]. For follow-up, regular assessments are recommended at 3-month intervals in the first year, followed by semiannual checks in the second year and annual evaluations for the subsequent two years. Various methods, such as ultrasound, X-rays, and liver function tests, are suggested to assess lymph nodes and multiple different organs [8].

In conclusion, MSAs are uncommon malignant adnexal skin tumors often originating from benign spiradenomas. Preventive excision of these tumors are considered due to their potential progression to MSA. Surgery is the primary treatment, emphasizing wide local excision with a minimum 1 cm margin to reduce metastasis risk. Close follow-up is crucial for early detection of recurrences or spread.
